# Lyme Disease Causing Complex Bullous Lesions: A Case Report

**DOI:** 10.7759/cureus.92859

**Published:** 2025-09-21

**Authors:** Carlos E Levischi

**Affiliations:** 1 Internal Medicine, Hospital Israelita Albert Einstein, São Paulo, BRA

**Keywords:** acquired epidermolysis bullosa, autoimmunity, borreliosis, erythema migrans, lyme disease

## Abstract

Lyme disease (LD), caused by the spirochete *Borrelia burgdorferi*, is a multisystemic zoonosis that can progress to severe autoimmune manifestations in its advanced stages. This article describes the case of a Brazilian patient who developed acquired epidermolysis bullosa (EBA) following an LD diagnosis. Initially, the patient presented with atypical erythema migrans, which later evolved into multiple bullous lesions characterized by cycles of exacerbation and remission. Histopathological investigation revealed subepidermal cleavage with linear IgG deposits along the basement membrane, confirming the diagnosis of EBA.

Treatment consisted of doxycycline and dapsone for 90 days, leading to regression of the lesions, normalization of inflammatory markers, and significant clinical improvement. After completing antibiotic therapy, immunomodulation with colchicine and hydroxychloroquine was initiated to control the autoimmune response. This case highlights the immune-mediated impact of LD, where autoantibodies, such as anti-collagen VII, can play a central role in perpetuating symptoms.

These findings emphasize the importance of a personalized diagnostic and therapeutic approach, incorporating both antibiotics and immunomodulators, to manage LD-associated autoimmune complications. The complexity of the disease underscores the necessity of multidisciplinary care and integrated interventions to minimize sequelae and improve the quality of life for affected patients.

## Introduction

Lyme disease (LD), also known as Lyme borreliosis, is a multisystemic zoonosis of global reach caused by infection with spirochetes from the *Borrelia burgdorferi *sensu lato group. Transmission occurs via *Ixodes ricinus* complex ticks, primarily in regions of the Northern Hemisphere. This condition has garnered increasing attention from the medical and scientific communities, not only due to its wide geographic distribution but also because of its significant impact on various organ systems. Its clinical complexity poses substantial challenges for both diagnosis and therapeutic management, necessitating a meticulous and interdisciplinary approach [1-3].

Clinically, LD progresses through distinct stages, each with specific characteristics. In the acute phase, the earliest and most characteristic manifestation is erythema migrans (EM), a hallmark cutaneous lesion indicating the initial infection. As the disease advances to the early disseminated stage, additional cutaneous lesions may appear, resembling or differing from those of the initial phase. This stage is also associated with systemic complications, including cardiac abnormalities, ocular involvement, and significant neurological manifestations such as meningitis, encephalitis, and cranial or peripheral neuropathies [1].

The advanced stage, known as late disseminated or chronic LD, occurs months or even years after the initial infection. During this phase, LD can impact multiple organ systems, presenting with diverse symptoms such as arthritis, joint pain, depression, cognitive impairments, and even psychiatric disorders. Diagnosing LD requires a multidimensional approach to ensure accuracy. It is crucial to evaluate characteristic clinical manifestations, such as EM and associated symptoms, alongside a compatible epidemiological history, such as exposure to endemic areas or reports of tick bites. Definitive diagnosis is achieved through laboratory testing, particularly positive serology for *Borrelia burgdorferi* using standardized and validated methods [2-4]. This comprehensive approach is essential to differentiate LD from other conditions with similar clinical presentations and to establish appropriate patient management.

In Brazil, research on LD began in the late 1980s, marking the start of national studies on this emerging condition [5]. In 1987, the first cases of cutaneous manifestations were described, highlighting EM as one of the disease's hallmark early signs [6,7]. Subsequently, in 1992, reports of Brazilian cases with systemic manifestations emerged, broadening the understanding of the disease's multisystemic impact in the national context [8].

Since then, new cases have been documented, allowing for the identification of clinical, laboratory, and epidemiological features unique to the Brazilian variant of the disease. These peculiarities differ in various aspects from the classic form of LD observed in the Northern Hemisphere, which points to the need for diagnostic and therapeutic approaches tailored to the local context [9,10].

The particularities identified in Brazilian cases have led to the definition of a distinct clinical entity, known as Brazilian Lyme-like disease or Brazilian borreliosis. Despite sharing characteristics of classic LD, such as EM and systemic complications, this variant exhibits significant peculiarities. Among these are the high frequency of clinical recurrences, the presence of immunological disorders, and the production of autoantibodies, which contribute to a more prolonged and predominantly immune-mediated disease progression [11,12].

This characterization underscores the relevance of the local epidemiological context and the need for diagnostic and therapeutic approaches tailored to the specificities of Brazilian Lyme-like disease, which can be found in all regions of the country [13].

## Case presentation

A 38-year-old Black female patient, born and residing in Rio de Janeiro, Brazil, living in an urban area near forested regions, presented with a history of hypothyroidism controlled with levothyroxine at a dose of 125 mcg, generalized anxiety disorder managed with desvenlafaxine at 50 mg/day, and alprazolam at 0.5 mg as a rescue medication. 

**Figure 1:**
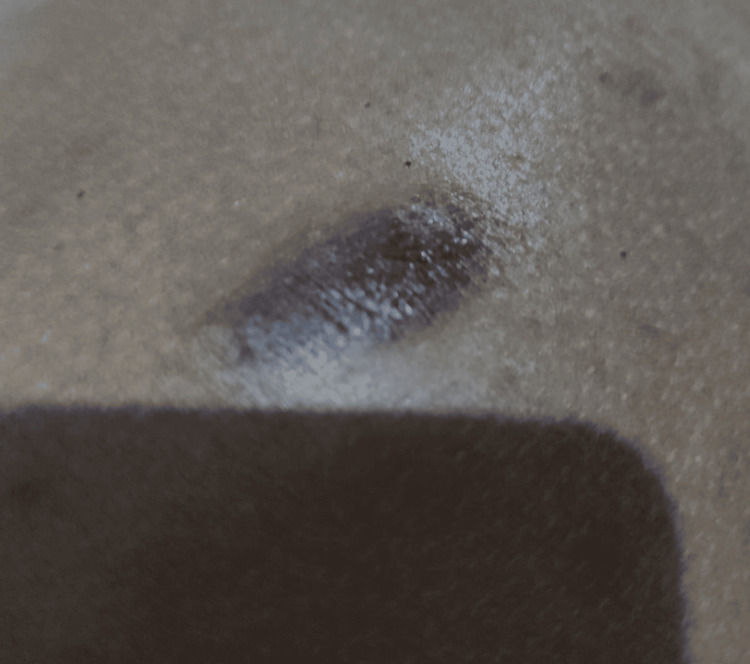
Hyperpigmented dorsal lesion, characteristic of atypical erythema migrans

In September 2023, the patient reported a tick bite on her dorsal region, which developed into a hyperpigmented lesion measuring approximately 5 cm. The lesion evolved slowly and was occasionally associated with local discomfort and pruritus (Figure 1). In January 2024, an incisional biopsy of the lesion was performed. 

**Figure 2:**
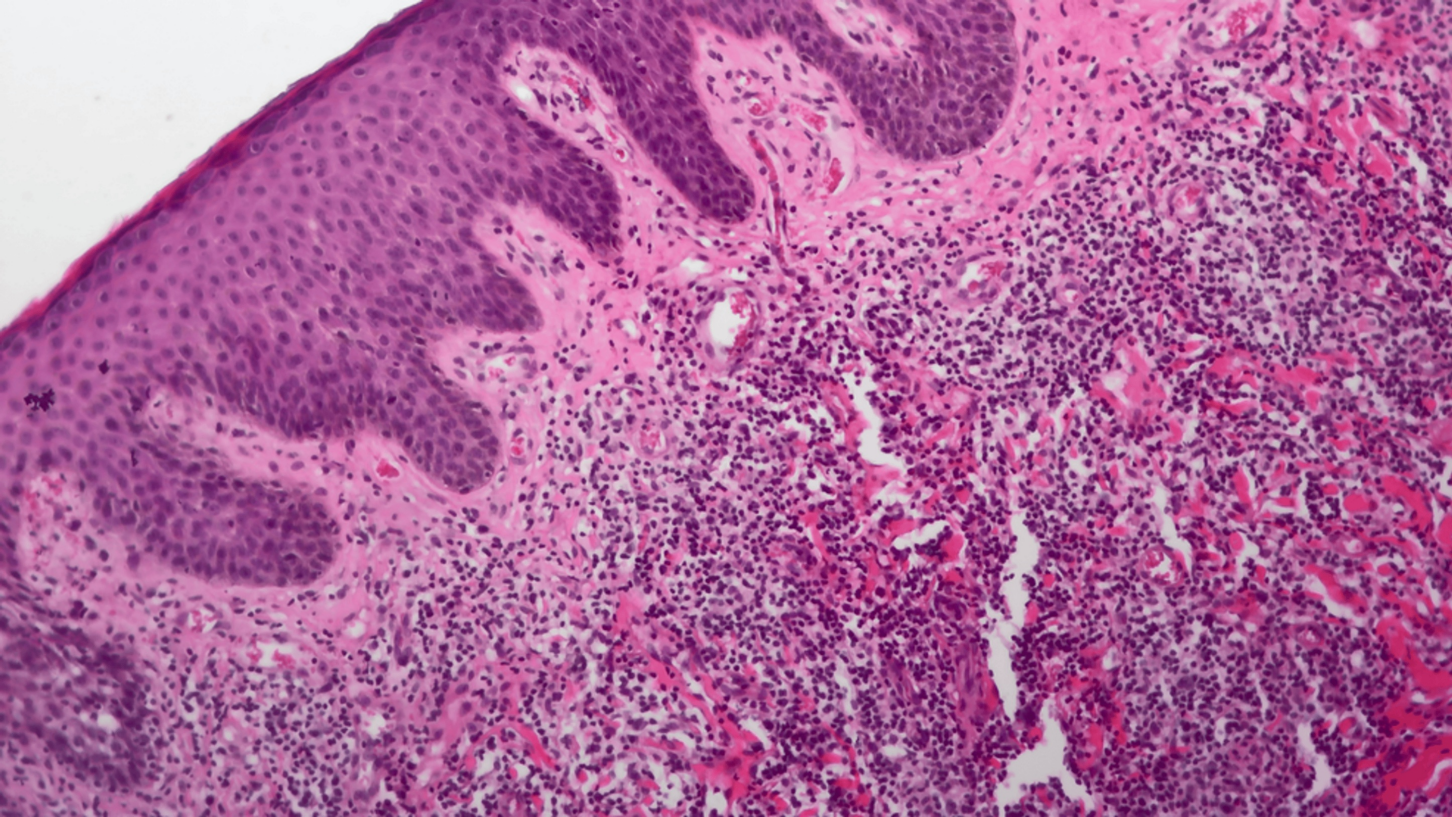
Histopathological examination of the lesion showing perivascular inflammatory infiltrate

The histopathological examination revealed a marked perivascular inflammatory infiltrate in the superficial and mid-dermis, predominantly lymphohistiocytic with numerous eosinophils (Figure 2). The report suggested the following: "Correlate with clinical findings to investigate arthropod bites (and mimickers) and chronic eczema." 

At the end of January 2024, the patient underwent blood tests for diagnostic investigation, which revealed mild anemia (Hb 10.9 g/dL; reference minimum 12 g/dL) and a leukogram and platelet count within normal limits. The erythrocyte sedimentation rate (ESR) was 90 mm/1 h (reference value <20 mm/1 h), and the C-reactive protein (CRP) level was 10 mg/L (reference value <5 mg/L). Serology tests for human immunodeficiency virus (HIV), human T-lymphotropic virus (HTLV), and hepatitis B and C were all negative. Further investigations, including immunoglobulin levels, urinary porphyrins, antinuclear factor (ANF), antineutrophil cytoplasmic antibodies (ANCA), rheumatoid factor, protein immunofixation, and protein electrophoresis, were all within normal ranges. Serological testing for LD revealed a positive IgM result via the enzyme-linked immunosorbent assay (ELISA) method, confirmed by Western blot. At that time, treatment with doxycycline 100 mg twice daily for 21 days was initiated, leading to the regression of the dorsal hyperpigmented lesion.

**Figure 3:**
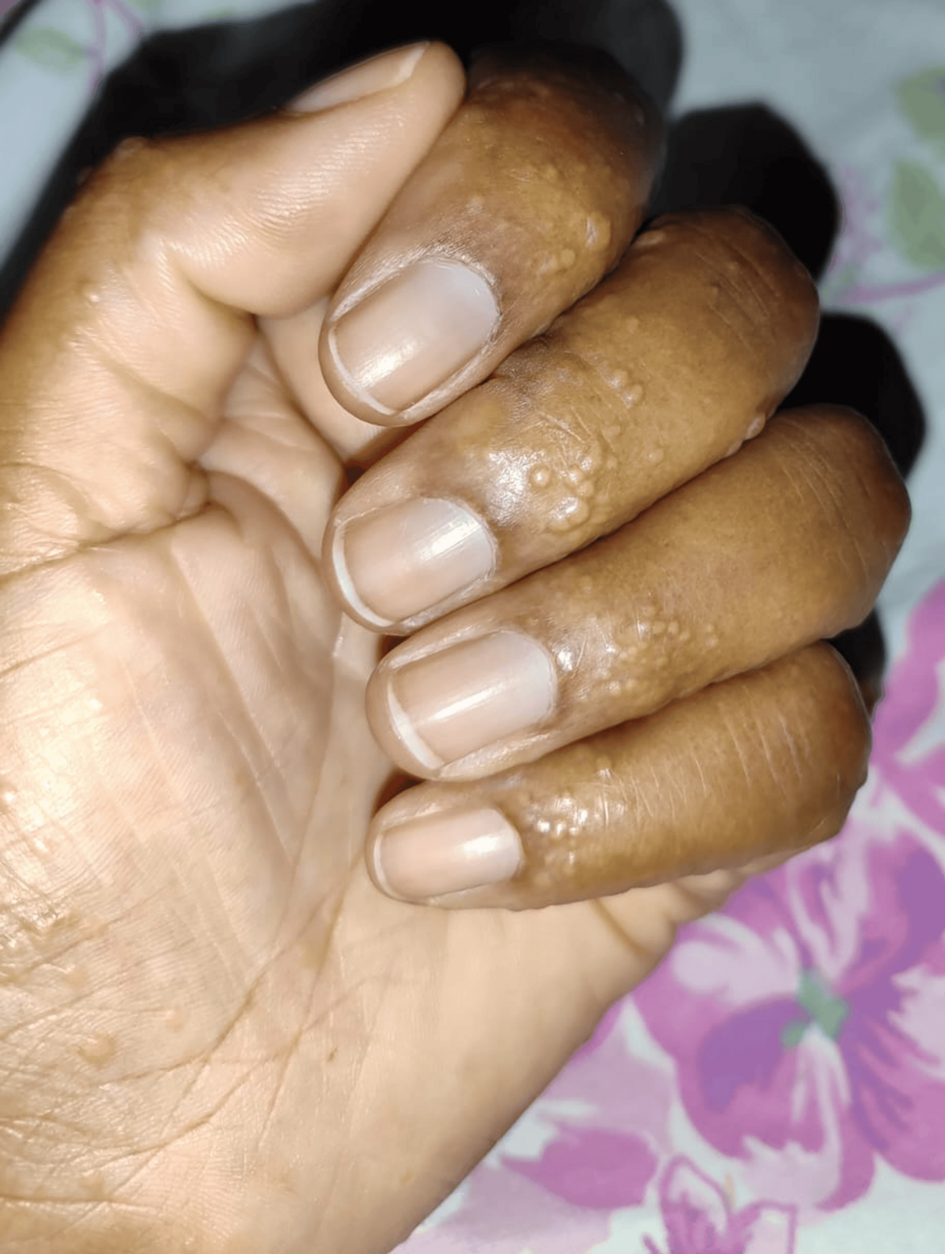
Multiple vesicular lesions on the hand, affecting both the palm and fingers

**Figure 4:**
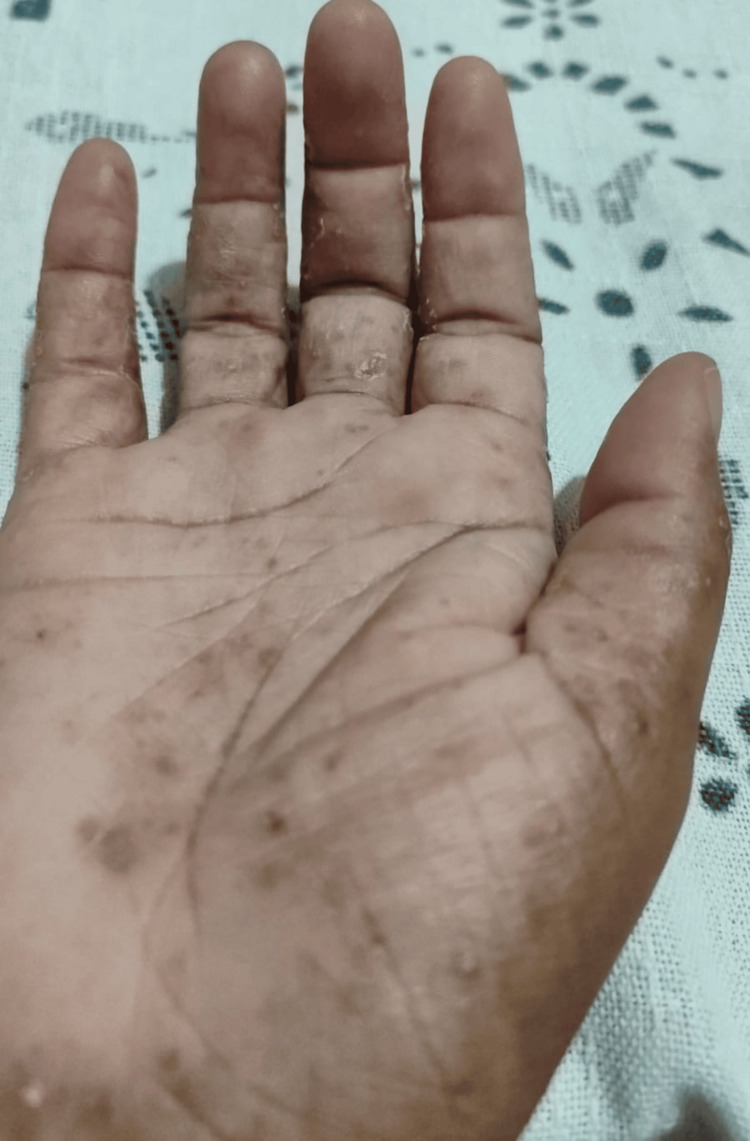
Remission of the lesions with the formation of atrophic and hyperpigmented scarring

**Figure 5:**
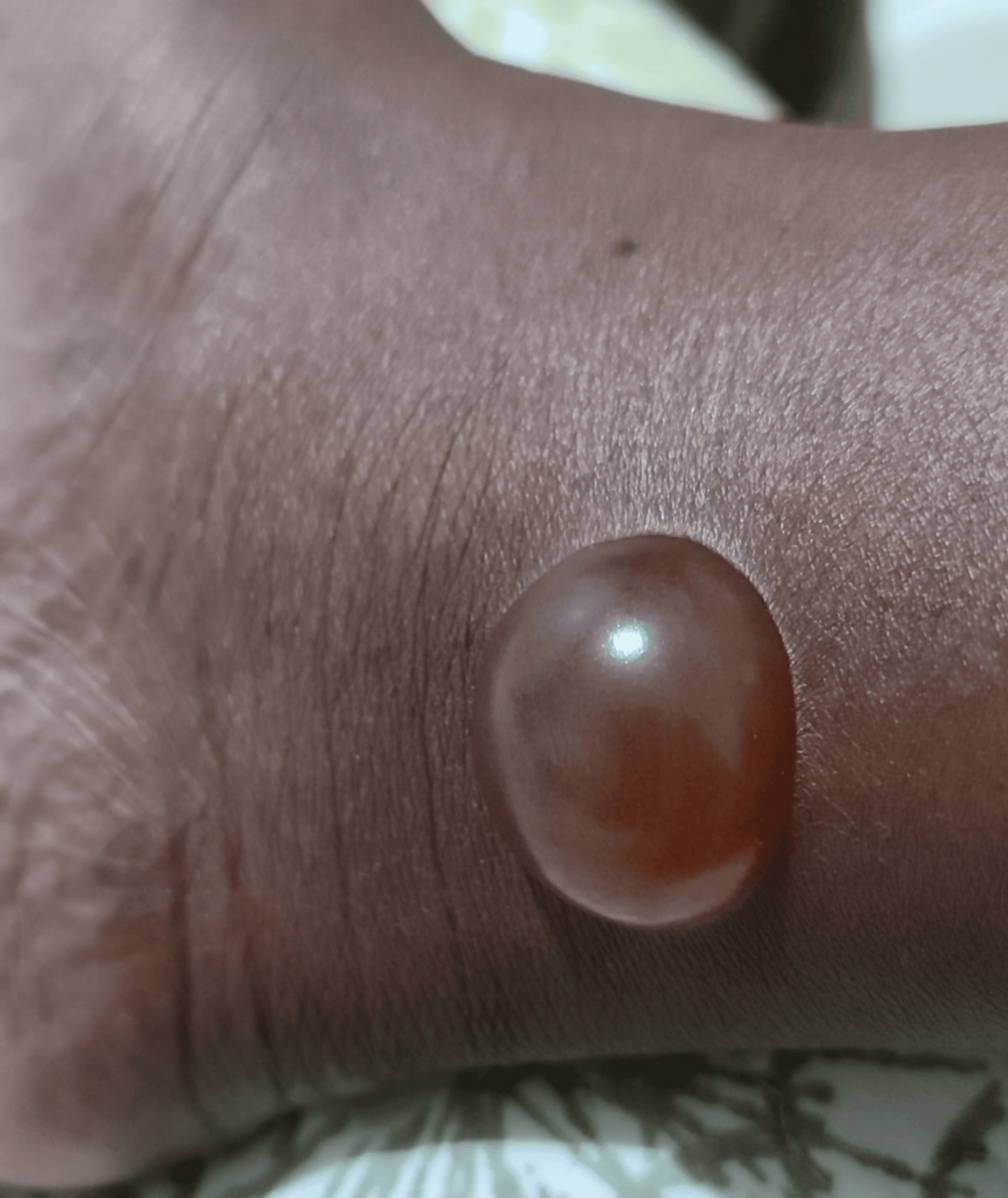
Bullous lesion on the ankle, with no visible inflammation in the surrounding skin

Starting in March 2024, the patient developed multiple vesicular lesions on both hands of variable size, tense, and containing clear fluid (Figure 3). These lesions followed a pattern of exacerbation and spontaneous remission, leaving behind atrophic and hyperpigmented scars (Figure 4). It was not uncommon for vesicles to appear in other body regions associated with trauma, such as the ankles (Figure 5). The patient also reported worsening anxiety and cognitive disturbances impairing reasoning, concentration, and memory. At this point, she also experienced significant fatigue, which interfered with her daily activities. Blood tests demonstrated persistent inflammatory activity, with the ESR at 42 mm/1 h (reference value <20 mm/1 h), indicating ongoing systemic inflammation. 

**Figure 6:**
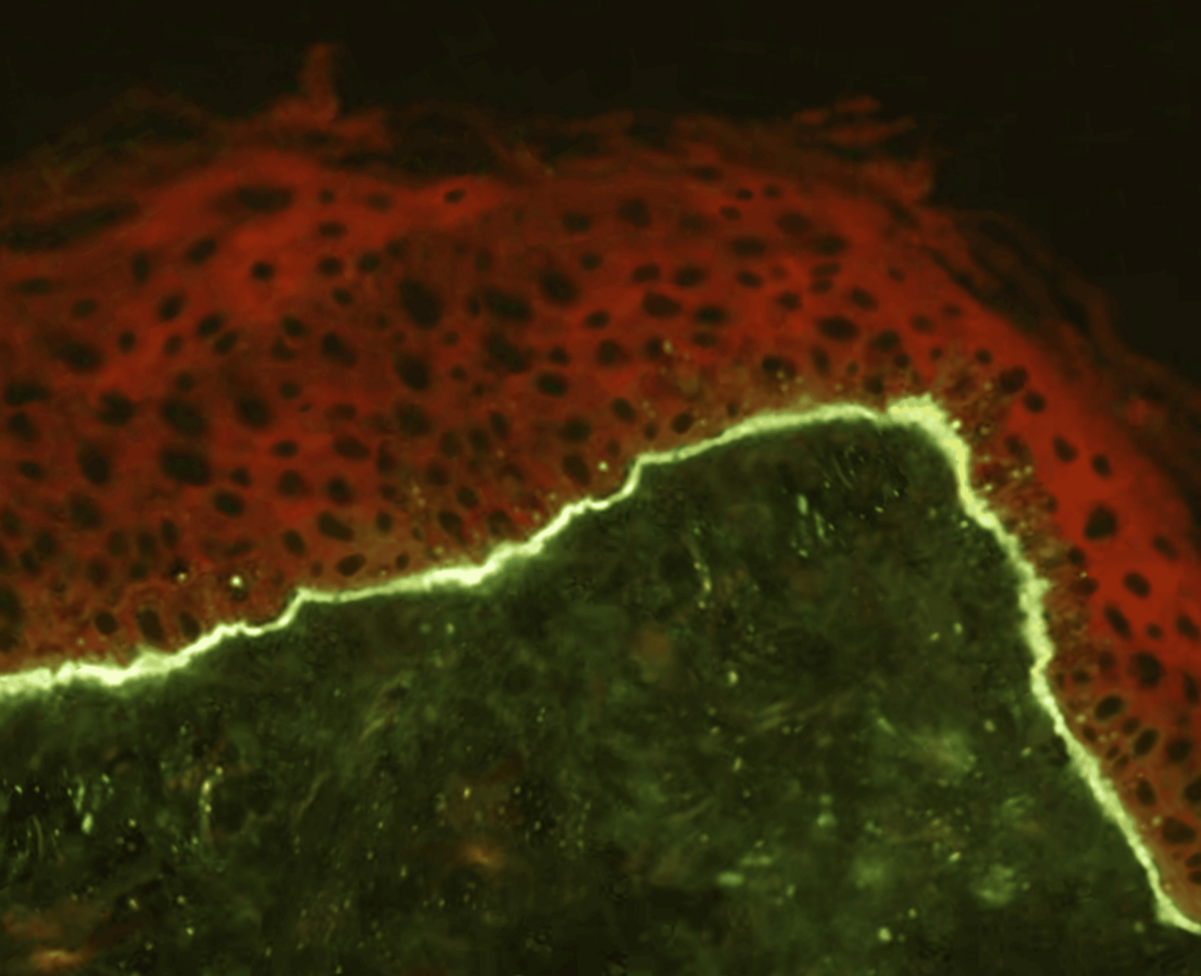
In direct immunofluorescence, linear deposition of IgG was observed in the basement membrane

A biopsy of a bullous lesion was performed, and histopathology revealed vesicular dermatitis with a vesicle formed by subepidermal cleavage, serous content containing few inflammatory cells, mild infiltrates, and vascular neogenesis at the base of the vesicle, findings consistent with acquired epidermolysis bullosa (EBA). Direct immunofluorescence demonstrated linear IgG deposits along the basement membrane (Figure 6). 

**Figure 7:**
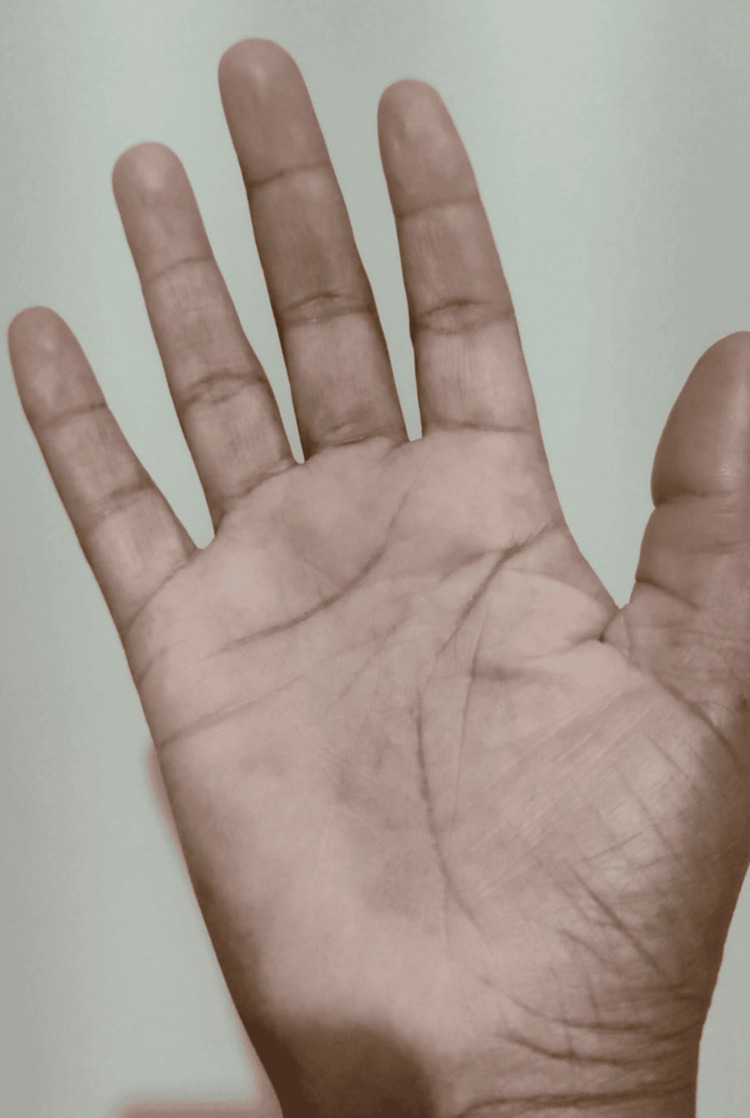
The treatment has resulted in the complete regression of the lesions

**Figure 8:**
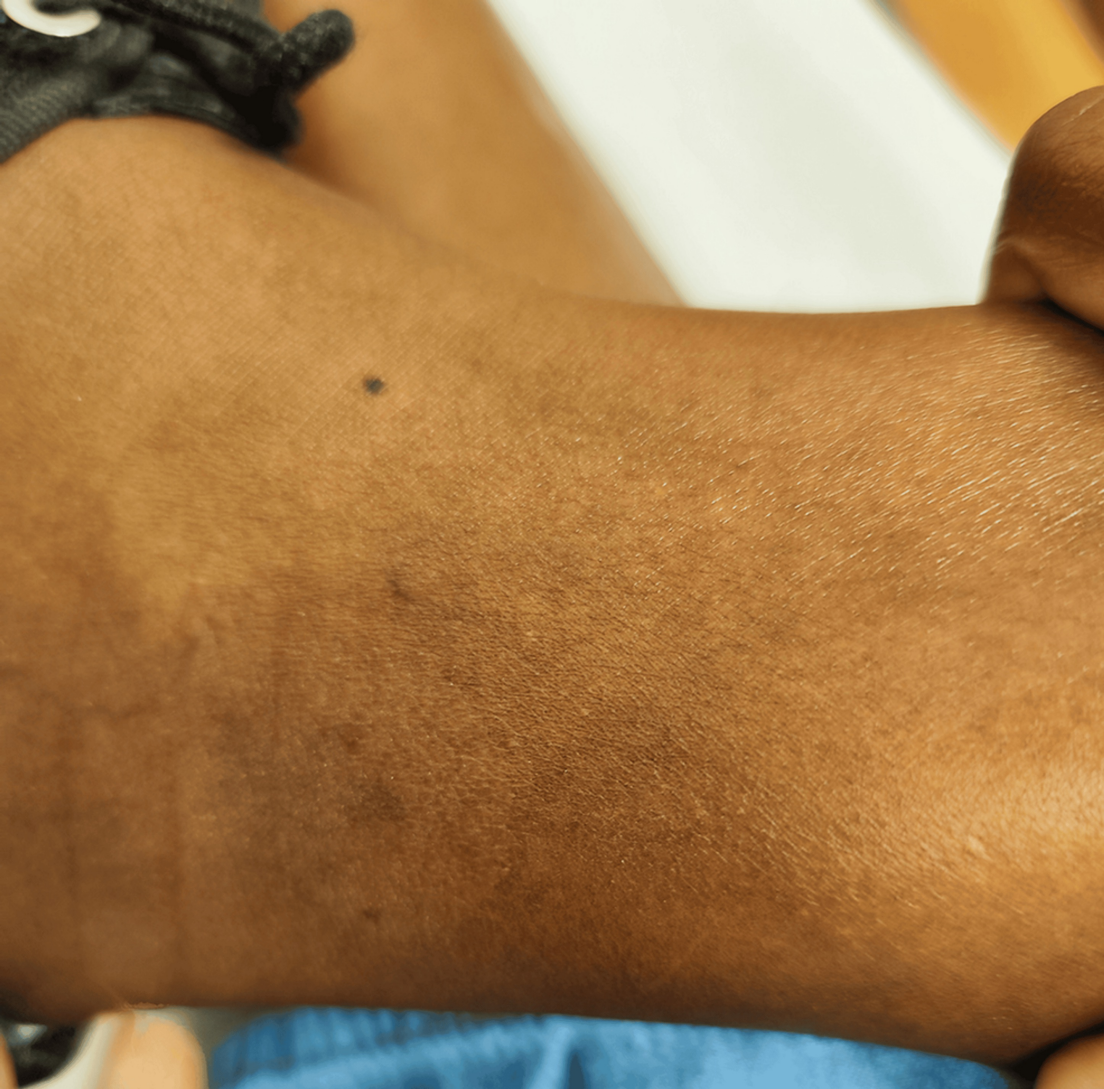
Complete regression of the lesions following the implemented treatment

In this case, the persistently elevated ESR supported the interpretation of an immune-mediated process, taking into account that Brazilian borreliosis in its chronic form rarely presents with detectable inflammatory activity in routine blood tests. The possibility of an autoimmune bullous dermatosis, compatible with acquired EBA, was considered. Based on the patient's clinical and laboratory findings, a more extensive treatment for Brazilian Lyme-like disease was undertaken, initiating doxycycline 100 mg twice daily and dapsone 50 mg twice daily. The regimen was maintained for a total of 90 days, resulting in the complete regression of the lesions (Figure 7 and Figure 8), normalization of inflammatory markers, and improvement in cognitive complaints, anxiety, and fatigue. The patient was monitored clinically and through laboratory tests, with no evidence of hepatic or renal dysfunction or worsening anemia during or after the treatment period.

Following the completion of antibiotic therapy, immunomodulation therapy was initiated with colchicine at a dose of 0.5 mg twice daily and hydroxychloroquine at 400 mg daily. This treatment is planned to continue for at least 12 months.

## Discussion

EM is the characteristic cutaneous lesion of LD in its early stage. The presence of *Borrelia burgdorferi* in EM lesions was first described by Berger et al. [14] and Steere et al. [15].

EM typically presents as a target-like skin lesion, initially appearing as a red or pink spot that gradually expands. In many cases, the central area of the lesion clears, creating an annular appearance. Although usually not painful, EM may cause mild itching or discomfort. It typically develops at the site of the tick bite, generally within 3-30 days of exposure. This type of lesion is widely regarded as the most common clinical marker of early-stage LD and tends to regress and resolve over time, even in untreated patients.

Some patients may present with atypical cutaneous lesions instead of the characteristic EM, including vesicular, scaling, or purpuric-hemorrhagic lesions [16]. Additionally, erythematous lesions with small satellite points, resulting from local hematogenous dissemination, may also be observed. In individuals of African descent, typical EM or even atypical lesions can be challenging to identify. A skin biopsy may be essential to exclude differential diagnoses such as erythema multiforme, lupus erythematosus, fungal infections, or granuloma annulare, thereby enabling accurate diagnosis [16].

Typical EM is histologically characterized by mild-to-moderate perivascular infiltrates predominantly composed of lymphocytes, with small amounts of plasma cells and mast cells. Vasculitis is not observed, nor is vascular proliferation. Collagen remains intact, and the infiltrate is confined to the immediate perivascular regions [17].

The main histopathological features of atypical cutaneous lesions in LD include superficial dermal and deep perivascular infiltrates, as well as interstitial infiltrates of varying intensity, primarily composed of lymphocytes but also containing some eosinophils and plasma cells. Endothelial exocytosis or vascular necrosis is absent, and skin adnexa are typically normal [18].

The pathogenesis of LD in its early stages is primarily driven by the presence of viable spirochetes of the *Borrelia* genus at the site of cutaneous inflammation, which triggers local immune responses. However, as the disease progresses to more advanced stages, clinical manifestations become largely attributed to autoimmune mechanisms that arise as a consequence of the initial infection.

This transition from a pathology directly mediated by infection to a predominantly autoimmune process illustrates the inherent complexity of LD and suggests that there must be a personalized approach to its diagnosis and management [19].

Infections are widely recognized as potential triggers for the development of autoimmune diseases in humans. An infectious agent may specifically activate lymphocytes directed toward the antigen while simultaneously providing a second, nonspecific signal essential to inducing a pathogenic adaptive immune response. This process, which combines specific activation with nonspecific costimulatory signals, is central to initiating autoimmune responses, contributing to immune system dysregulation and perpetuation of related pathological states [20].

Autoimmunity induced by foreign antigens can occur through various mechanisms, including molecular mimicry, epitope spreading, bystander activation, and polyclonal activation of B and T cells, often triggered by the presence of persistent pathogens and chronic inflammation. Among these mechanisms, molecular mimicry is the most extensively studied in relation to LD. This process arises when there is a high structural similarity between *Borrelia *antigens and the host's self-antigens, leading to cross-reactivity. Such cross-reactivity can involve both T cells and antibodies, directing immune responses against the host's own tissues, thereby contributing to the persistence of autoimmune symptoms and disease progression [21].

Autoimmunity induced by *Borrelia *infection is the leading hypothesis regarding the persistence of symptoms in the late stages of LD. To date, various autoantigens have been identified as potential targets of both B- and T-cell responses [22] in patients with chronic symptoms, including heat shock proteins (hsp), myosin, myelin basic protein (MBP), human leukocyte function-associated antigen-1 (hLFA-1), endothelial cell growth factor (ECGF) [23], apolipoprotein B100 [24], annexin A2 [25], matrix metalloproteinase-10 [26], human cytokeratin 10 [27], and, more recently, gamma-enolase [28]. Although antibodies against these autoantigens may appear during the early stages of infection, they are typically non-pathogenic at that point, showing little to no significant associated T-cell activation. However, as the disease progresses, particularly into its later stages, these autoantibodies are often accompanied by robust T-cell responses. This interaction suggests an amplification of the autoimmune process, contributing to the persistence of symptoms and the characteristic complications of advanced LD [22-25,27].

A study identified various autoantigens with cross-reactivity, recognized as ligands by T cells specific to *Borrelia *proteins. While cross-reactivity was found to be relatively common, the results indicated that its mere presence does not necessarily lead to the development of an autoimmune pathology. These findings suggest that additional factors, such as the inflammatory context or the host's genetic predisposition, are required for this reactivity to translate into clinical disease [29].

The control of *Borrelia burgdorferi* infection in humans is largely mediated by innate and adaptive immune responses, particularly the role of type 1 helper T (TH1) cells. These responses are critical for containing the infection; however, excessive levels of inflammatory mediators associated with TH1 activation may contribute to more severe forms of LD, including more symptomatic early infections and cases of antibiotic-refractory Lyme arthritis [22-24].

Additionally, an exacerbated TH17 immune response, observed primarily in the early phases of late-stage LD, has been correlated with the development of autoantibodies. This immune profile also plays a significant role in the progression to antibiotic-refractory Lyme arthritis, which emphasizes the value of maintaining a precise balance in immune responses to prevent severe complications [22].

Both the genetic factors of *Borrelia burgdorferi* and those of the host play a crucial role in inducing autoimmune responses associated with LD. For instance, Bb RST1 strains, which account for approximately 30-50% of infections in the northeastern United States, exhibit a more pronounced inflammatory profile compared to other bacterial variants. This heightened inflammatory capacity of Bb RST1 strains is directly linked to a greater frequency of antibiotic-refractory Lyme arthritis cases, illustrating the importance of the bacterial genotype and its interaction with the host's immune system in determining disease severity [23].

However, our current understanding represents only a fraction of the spectrum of autoimmune complications that LD can trigger. The clinical case presented here involved a progression to a condition resembling autoimmune-acquired EBA.

Autoimmune blistering dermatoses are defined by vesicles and blisters as primary cutaneous manifestations. These lesions are classified based on the location of the blister relative to the skin layers, either intraepidermal or subepidermal. EBA is a rare autoimmune condition caused by the production of autoantibodies targeting type VII collagen, a crucial component of the anchoring fibrils in stratified squamous epithelium. The interaction between these autoantibodies and type VII collagen initiates a complex inflammatory response that disrupts the adhesion between the dermis and epidermis, resulting in blister formation on the skin and mucous membranes.

The mechanobullous form of EBA is characterized by cutaneous fragility, tense blisters, erosions, and milia, particularly in areas subjected to mechanical trauma. These clinical features reflect the profound impact of immune-mediated responses on the skin's structure and integrity [30].

EBA is characterized by the formation of tense blisters, erosions, and marked cutaneous fragility. These blisters, originating from the lower part of the basement membrane zone, tend to persist for several days and may contain clear or hemorrhagic fluid. In the mechanobullous form of EBA, vesiculobullous lesions and cutaneous fragility predominantly occur in areas subjected to pressure or repeated trauma, such as the extensor surfaces of acral regions, including the hands, feet, elbows, knees, and pretibial area.

These lesions typically appear on normal skin, without prior signs of edema or erythema, and often develop rapidly following minimal trauma, sometimes within hours. Mucosal lesions are common and can significantly impair patients' quality of life. Over the course of the disease, additional complications may arise, such as the formation of milia, atrophic scarring, pigmentary changes (hyper- or hypopigmentation), nail dystrophy and loss, cicatricial alopecia, and even digital contractures. These chronic and debilitating manifestations highlight the severe and enduring impact of the condition [31].

EBA has been associated with various systemic conditions, including amyloidosis, thyroiditis, multiple endocrine syndrome, rheumatoid arthritis, pulmonary fibrosis, chronic lymphocytic leukemia, thymoma, and diabetes mellitus. However, most of these associations are based on isolated case reports, lacking consistent evidence to establish them as part of the disease's pathogenesis. The only well-established and widely recognized association is with inflammatory bowel disease (IBD), particularly Crohn's disease. This connection highlights the potential underlying immunological interactions between EBA and chronic inflammatory conditions, emphasizing the importance of investigating comorbidities in patients diagnosed with EBA [32].

Histopathological examination plays a pivotal role in the diagnosis of EBA, typically revealing subepidermal blisters without significant inflammatory components. Direct immunofluorescence is an essential diagnostic method, demonstrating linear deposits of IgG and C3 along the basement membrane zone, with IgA and IgM occasionally observed. The predominant pathogenic autoantibodies belong to the IgG class, and their detection is critical for diagnostic confirmation. Indirect immunofluorescence (IIF), although positive in only approximately 50% of cases, can be enhanced by the "Salt Split Skin" technique. This method significantly increases the sensitivity of IIF by identifying linear deposition of immunoglobulins at the dermal level, making it particularly valuable in cases with atypical clinical manifestations or inconclusive initial results [33].

The primary goals of EBA treatment are to control disease activity and prevent both recurrences and permanent sequelae. Therapeutic success is defined by the cessation of new lesion formation and the complete healing of existing ones. However, there is no single, specific therapy for EBA, and treatment responses vary significantly among patients.

Generally, children tend to achieve complete disease remission, whereas adults, particularly those with mucosal involvement, face a more reserved prognosis. In mild cases, systemic corticosteroids and immunomodulators such as colchicine and dapsone are often sufficient to control the disease. In more severe cases, it becomes necessary to combine corticosteroid therapy with more potent immunosuppressants, intravenous immunoglobulin, and biologic agents like rituximab. These strategies underscore the need for a personalized approach to effectively manage the severity and complications of EBA [34].

Regarding the therapeutic choice for the presented case, dapsone (4,4-diaminodiphenylsulfone) belongs to the sulfone class, historically widely used as antimicrobials. It is considered a bacteriostatic antimicrobial agent. In dermatology, however, its use is primarily driven by its immunomodulatory effects. Dapsone inhibits neutrophil adhesion and reduces IL-8, a key mediator of neutrophil-driven inflammation. Additionally, the adhesion of IgA is inhibited by this drug [35].

Dapsone's efficacy as an antibiotic against *Borrelia *has been demonstrated in vitro [36], in animal models [37], and in humans [38]. Due to its dual effect on both pathologies, it was selected as one of the drugs for treatment. The second medication, doxycycline, was an obvious alternative due to its established role as the first-line treatment for LD in adults.

Combination therapy is an alternative approach for the treatment of *Borrelia burgdorferi* infection and is well-established for managing persistent bacteria such as *Mycobacterium tuberculosis* and *Brucella melitensis* in both humans and animal models [37]. The patient's favorable response strengthens the appropriateness of this therapeutic choice, particularly considering a prior monotherapy with doxycycline had failed to yield satisfactory results. Extended antibiotic treatments have also been shown to be effective in patients with persistent LD symptoms [39], increasingly emerging as a viable option for individuals infected with the Brazilian variant of the disease who experience chronic, hard-to-resolve symptoms.

## Conclusions

LD, characterized as a multisystemic infection caused by *Borrelia burgdorferi*, presents complex clinical manifestations that progress over time, including EM, arthritis, neuropathies, and autoimmune complications. Recent studies highlight the transition from an initial infectious response to autoimmune mechanisms in the advanced stages of the disease, as evidenced by the production of autoantibodies targeting human antigens. These autoantibodies have been implicated in the pathogenesis of chronic manifestations, underscoring the immune-mediated impact of the infection.

The clinical case of a patient presenting with a condition resembling EBA exemplifies the relevance of autoimmune complications associated with LD, particularly in its late stage. The presence of anti-collagen VII autoantibodies, leading to skin fragility and blistering lesions, emphasizes the central role of autoimmunity in sustaining symptoms even after initial bacterial control. These findings highlight the importance of a personalized approach, including immunomodulatory therapies, to manage these complications and prevent permanent sequelae.

Thus, LD transcends being a simple bacterial infection, representing a condition that challenges both diagnosis and therapeutic management due to its complex interplay of infectious and autoimmune factors. Multidisciplinary follow-up and integrated therapies are essential to address its wide range of clinical manifestations and to ensure an improved quality of life for patients.

## References

[1] Steere AC (2001). Lyme disease. N Engl J Med.

[2] Steere AC, Batsford WP, Weinberg M, Alexander J, Berger HJ, Wolfson S, Malawista SE (1980). Lyme carditis: cardiac abnormalities of Lyme disease. Ann Intern Med.

[3] Steere AC, Gross D, Meyer AL, Huber BT (2001). Autoimmune mechanisms in antibiotic treatment-resistant Lyme arthritis. J Autoimmun.

[4] Yoshinari NH, Barros PJ, Gauditano G, Costa IP, Fonseca AH, Bonoldi VL (2000). Report of 57 cases of Lyme-like disease (LLD) in Brazil. Arthritis Rheum.

[5] Yoshinari NH, Steere AC, Cossermelli W (1989). A review of Lyme disease [Article in Portuguese]. AMB Rev Assoc Med Bras.

[6] Talhari S, Schettini AP, Parreira VJ (1987). Eritema crônico migrans/doença de Lyme-estudo de três casos. Anais do Congresso Brasileiro de Dermatologia.

[7] Talhari S, Talhari AC, Ferreira LC (1992). Eritema cronicum migrans, eritema migratório, doença de Lyme ou borreliose de Lyme. An Bras Dermatol.

[8] Yoshinari NH, Barros PJ, Cruz FC (1992). Clínica e sorologia da doença de Lyme no Brasileiro. Rev Bras Reumatol.

[9] Costa IP, Bonoldi VL, Yoshinari NH (2001). Perfil clínico e laboratorial da doença de Lyme-símile no Estado de Mato Grosso do Sul: análise de 16 pacientes. Rev Bras Reumatol.

[10] Yoshinari NH, de Barros PJ, Bonoldi VL (1997). Outline of Lyme borreliosis in Brazil [Article in Portuguese]. Rev Hosp Clin Fac Med Sao Paulo.

[11] Mantovani E, Costa IP, Gauditano G, Bonoldi VL, Higuchi ML, Yoshinari NH (2007). Description of Lyme disease-like syndrome in Brazil. Is it a new tick borne disease or Lyme disease variation?. Braz J Med Biol Res.

[12] Yoshinari NH, Barros PJ, Fonseca AH, Bonoldi VL, Battesti DM, Schumaker TT, Cossermelli W (1995). Borreliose de Lyme: zoonose emergente de interesse multidisciplinar. Newslab.

[13] Levischi CE Jr (2023). Facial paralysis due to Brazilian Lyme disease: a case report and literature review. Cureus.

[14] Berger BW, Clemmensen OJ, Ackerman AB (1983). Lyme disease is a spirochetosis. A review of the disease and evidence for its cause. Am J Dermatopathol.

[15] Steere AC, Grodzicki RL, Kornblatt AN (1983). The spirochetal etiology of Lyme disease. N Engl J Med.

[16] Asbrink E, Hovmark A (1988). Early and late cutaneous manifestations in Ixodes-borne borreliosis (erythema migrans borreliosis, Lyme borreliosis). Ann N Y Acad Sci.

[17] Duray PH, Steere AC (1988). Clinical pathologic correlations of Lyme disease by stage. Ann N Y Acad Sci.

[18] Wu YS, Zhang WF, Feng FP, Wang BZ, Zhang YJ (1993). Atypical cutaneous lesions of Lyme disease. Clin Exp Dermatol.

[19] Yehudina Y, Trypilka S (2021). Lyme borreliosis as a trigger for autoimmune disease. Cureus.

[20] Rose NR (2008). The adjuvant effect in infection and autoimmunity. Clin Rev Allergy Immunol.

[21] Adkison H, Embers ME (2023). Lyme disease and the pursuit of a clinical cure. Front Med (Lausanne).

[22] Strle K, Sulka KB, Pianta A (2017). T-helper 17 cell cytokine responses in Lyme disease correlate with Borrelia burgdorferi antibodies during early infection and with autoantibodies late in the illness in patients with antibiotic-refractory Lyme arthritis. Clin Infect Dis.

[23] Drouin EE, Seward RJ, Strle K (2013). A novel human autoantigen, endothelial cell growth factor, is a target of T and B cell responses in patients with Lyme disease. Arthritis Rheum.

[24] Crowley JT, Drouin EE, Pianta A, Strle K, Wang Q, Costello CE, Steere AC (2015). A highly expressed human protein, apolipoprotein B-100, serves as an autoantigen in a subgroup of patients with Lyme disease. J Infect Dis.

[25] Pianta A, Drouin DE, Arvikar S (2014). Identification of annexin A2 as an autoantigen in rheumatoid arthritis and in Lyme arthritis. Arthritis Rheum.

[26] Crowley JT, Strle K, Drouin EE (2016). Matrix metalloproteinase-10 is a target of T and B cell responses that correlate with synovial pathology in patients with antibiotic-refractory Lyme arthritis. J Autoimmun.

[27] Ghosh S, Seward R, Costello CE, Stollar BD, Huber BT (2006). Autoantibodies from synovial lesions in chronic, antibiotic treatment-resistant Lyme arthritis bind cytokeratin-10. J Immunol.

[28] Maccallini P, Bonin S, Trevisan G (2018). Autoimmunity against a glycolytic enzyme as a possible cause for persistent symptoms in Lyme disease. Med Hypotheses.

[29] Maier B, Molinger M, Cope AP (2000). Multiple cross-reactive self-ligands for Borrelia burgdorferi-specific HLA-DR4-restricted T cells. Eur J Immunol.

[30] Miyamoto D, Gordilho JO, Santi CG, Porro AM (2022). Epidermolysis bullosa acquisita. An Bras Dermatol.

[31] Koga H, Prost-Squarcioni C, Iwata H, Jonkman MF, Ludwig RJ, Bieber K (2018). Epidermolysis bullosa acquisita: the 2019 update. Front Med (Lausanne).

[32] Gupta R, Woodley DT, Chen M (2012). Epidermolysis bullosa acquisita. Clin Dermatol.

[33] Fernandes MD, Chiminazzo MD, Tebcherani AJ, Aoki V, Sanchez AP (2009). Inflammatory epidermolysis bullosa acquisita: case report. An Bras Dermatol.

[34] Kridin K, Ahn C, Huang WC, Ansari A, Sami N (2019). Treatment update of autoimmune blistering diseases. Dermatol Clin.

[35] Ghaoui N, Hanna E, Abbas O, Kibbi AG, Kurban M (2020). Update on the use of dapsone in dermatology. Int J Dermatol.

[36] Horowitz RI, Murali K, Gaur G, Freeman PR, Sapi E (2020). Effect of dapsone alone and in combination with intracellular antibiotics against the biofilm form of B. burgdorferi. BMC Res Notes.

[37] Alruwaili Y, Jacobs MB, Hasenkampf NR, Tardo AC, McDaniel CE, Embers ME (2023). Superior efficacy of combination antibiotic therapy versus monotherapy in a mouse model of Lyme disease. Front Microbiol.

[38] Horowitz RI, Freeman PR (2020). Efficacy of double-dose dapsone combination therapy in the treatment of chronic Lyme disease/post-treatment Lyme disease syndrome (PTLDS) and associated co-infections: a report of three cases and retrospective chart review. Antibiotics (Basel).

[39] Stricker RB (2007). Counterpoint: long-term antibiotic therapy improves persistent symptoms associated with Lyme disease. Clin Infect Dis.

